# The Prevalence of Vitamin A Deficiency and Associated Factors in Pregnant Women Receiving Prenatal Care at a Reference Maternity Hospital in Northeastern Brazil

**DOI:** 10.3390/nu10091271

**Published:** 2018-09-09

**Authors:** Sabina Bastos Maia, Maria de Fátima Costa Caminha, Suzana Lins da Silva, Alex Sandro Rolland Souza, Camila Carvalho dos Santos, Malaquias Batista Filho

**Affiliations:** 1 Maternal and Child Healthcare Postgraduate Program, Instituto de Medicina Integral, Prof.Fernando Figueira (IMIP), 50070-550 Recife, Pernambuco, Brazil; fatimacaminha@imip.org.br (M.d.F.C.C.); suzanalinsilva@gmail.com (S.L.d.S.); alexrolland@uol.com.br (A.S.R.S.); malaquias.imip@gmail.com (M.B.F.); 2 Department of Obstetrics and Gynecology, Lauro Wanderley University Hospital, Federal University of Paraíba (UFPB), 58059-900 João Pessoa, Paraíba, Brazil; 3 Coordinator, Nursing Mentoring Program, Faculdade Pernambucana de Saúde (FPS), 51180-001 Recife, Pernambuco, Brazil; 4 Department of Maternal and Child Healthcare, Federal University of Pernambuco (UFPE), 50670-901 Recife, Pernambuco, Brazil; 5 Biological and Health Sciences Center, Catholic University of Pernambuco (UNICAP), 50050-900 Recife, Pernambuco, Brazil; 6 Coordinator, Research Department, IMIP, 50070-550 Recife, Pernambuco, Brazil; camilacarvalhoupe@gmail.com

**Keywords:** vitamin A deficiency, pregnancy, prevalence, risk factors

## Abstract

Vitamin A is essential for mother and child; however, vitamin A deficiency (VAD) remains a public health issue in various countries, affecting around 19 million pregnant women. In Brazil, the scarcity and inconsistency of data have prevented the prevalence and epidemiological status of VAD from being established. This study aimed to analyze vitamin A nutritional status in women receiving prenatal care at a reference center in northeastern Brazil. A cross-sectional study was conducted with a sample of 676 women. Serum retinol was measured by high-performance liquid chromatography. Subclinical infection was detected by measuring C-reactive protein (CRP). The World Health Organization criteria were used in the prevalence analysis, VAD classification level, and CRP effect evaluation. The prevalence of VAD (serum retinol <0.70 μmol/L) was 6.2% (95% confidence interval 4.5–8.3). In the univariate analysis, the variables significantly associated with VAD (*p* < 0.05) were having <12 years of schooling, being in the third trimester of pregnancy, and anemia. In the final multivariate model, the variables that remained significantly associated (*p* < 0.05) were being in the third trimester of pregnancy and anemia. VAD constituted a mild public health problem in this sample of pregnant women and was associated with the third trimester of pregnancy and maternal anemia.

## 1. Introduction

Vitamin A has multiple systemic functions. It is associated with bone development, exerts a protective effect on skin and mucosal integrity, plays an essential role in the functional capacity of the reproductive organs, participates in strengthening the immune system, acts in the development and maintenance of epithelial tissue, and contributes to the normal development of teeth and hair [1,2,3]. Adequate vitamin A levels are also indispensable for normal embryonic development [4], since the presence of severe vitamin A deficiency (VAD) in early pregnancy has been associated with malformations [5]. Humans and other vertebrates are unable to synthesize this lipid-soluble micronutrient; therefore, it has to be obtained from the diet. Vitamin A originates from two main sources: preformed vitamin A (retinol and retinyl ester) and provitamin A (beta-carotene) [6,7].

Vitamin A also plays a role in the morphology and physiology of the eyeball, by acting on cell differentiation and by guaranteeing the tissue integrity of the eyeball complex formation, particularly in its anterior segment (sclera and cornea) and the fundus of the eye (retina) with its singular functions of cones and rods [3]. VAD encompasses a set of eye signs and signals, with xerophthalmia being used as an umbrella term for the clinical spectrum of its ocular manifestations [8]. VAD is still considered the principal cause of avoidable blindness in the world [9]. Moreover, VAD is a predisposing and aggravating factor for comorbidities such as diarrhea, respiratory infections, measles, whooping cough, moderate and severe forms of malnutrition in children, and different dermatological lesions [3]. Attention has been increasingly drawn to the interaction between infections and VAD [10,11], with more data becoming available from experimental research in laboratory animals, as well as clinical and epidemiological studies. The findings of these studies should be incorporated into the approach and management used in healthcare, particularly in the case of pregnant women. In this respect, the study conducted by Christian et al. in 1998 [12] on the etiopathogenesis of nutritional morbidities, as well as their symptoms and outcomes, particularly in children and pregnant women, is extremely illustrative.

Although VAD has been identified as one of three health priorities worldwide in terms of micronutrient deficiencies (the other two being anemia and iodine deficiency), the epidemiological problem remains unsolved [13]. Conceptually, VAD has been shown to affect all age groups, from children to the elderly, including the fetal stage. Nevertheless, children and women of reproductive age, particularly pregnant and breast-feeding women, represent the groups that are most at risk, more specifically those living in the poorer regions of the world such as South Asia, Equatorial Africa, the Indonesian archipelago, Latin America, and the Caribbean [9]. VAD is estimated to affect around 19 million pregnant women, according to the World Health Organization (WHO), with 9.8 million being affected by night blindness [9].

The fact that specific nutritional deficiencies, which could easily be controlled through low-cost, highly effective interventions [14], still persist as endemic diseases to the present day, with some even reappearing or intensifying, would appear paradoxical. From a purely biological viewpoint, the cause of the problem insofar as the pregnant woman and her fetus are concerned is simple: the increased demand for vitamin A during pregnancy [15]. As elaborated further in this paper, it appears unjustifiable that irrespective of the notable advances made in combatting the more generic problems related to diet and nutrition, the country has no baseline data on the status of pregnant women in a setting in which 95% of such women receive prenatal care.

Alongside that simple biological equation, a multitude of complex internal and external factors contribute to the problem. Population-based evaluations of VAD in pregnant women in developing countries such as Brazil and other Latin American nations have often been hindered by inconsistent data. Indeed, data from field surveys conducted over the past 25 years have been questioned due to their small sample sizes, the selection of indicators, and the methods used in data collection and analysis [16,17,18]. However, the relatively high costs involved have prevented more accurate evaluations from being made. Nevertheless, in view of the importance of the subject, population-based studies must be conducted to assess the prevalence of VAD, at least in the most vulnerable populations (i.e., pregnant and breastfeeding women), as a means of defining the epidemiological status of the problem and its causes. The use of such data as a baseline status may ultimately identify strategies for intervention.

According to a literature review, data from descriptive and analytical evaluations remain insufficient for this task. Therefore, the objective of the present study was to evaluate vitamin A status in pregnant women receiving care at a reference maternal and child healthcare clinic in northeastern Brazil, a region recognized as the single largest concentration of poverty in Latin America.

## 2. Materials and Methods 

### 2.1. Study Design

This was a population-based, cross-sectional study using primary data from a survey entitled “The nutritional status of pregnant women: methodological and epidemiological aspects and implications in prenatal care”, conducted by the research group on nutrition at the Instituto de Medicina Integral Prof. Fernando Figueira (IMIP) and by the Department of Nutrition, Federal University of Pernambuco (UFPE). The objective of the original project was to describe the nutritional status (anemia, vitamin A deficiency, and classification of nutritional status) of pregnant women undergoing prenatal care. The participants of that study came from three different geographical regions of the state of Pernambuco: the coastal zone (Recife), a forested zone (Vitória de Santo Antão), and a semi-arid zone (Caruaru). In Recife, the sample consisted of women attending the Women’s Healthcare Center at IMIP where a range of services (primary care, specialist outpatient clinics, and a tertiary hospital) is available. Data collection was performed between September 2011 and April 2012.

### 2.2. Participants

For this study on vitamin A status, the target population consisted of pregnant women from the metropolitan area of Recife receiving prenatal care at IMIP. Pregnant women of 15 to 45 years of age were included. Women with kidney disease, liver disease, steatorrhea, or psychosis were excluded from the study, as were any cases of maternal Rh-isoimmunization.

The WHO criteria for VAD in pregnancy were used to calculate the sample size: VAD is defined as serum retinol levels <0.70 μmol/L, and VAD is considered a severe public health issue when its prevalence reaches 20% or more of the population of pregnant women [9,14]. Sample size was thus defined according to the following formula: $n=\frac{p\left(1-p\right)}{e^{2}}$, in which *p* is 18% (estimated prevalence) and the standard error of the estimate is 2%. The sample required was initially calculated as 369. Since the sample was not randomized, this number was increased by 20%, resulting in a total of 443. Finally, an additional 50% was added to that number to enable the sample to be stratified in a multivariate analysis, thus reaching a total required sample size of 665 pregnant women. The final study sample consisted of 676 pregnant women.

### 2.3. Evaluation of Serum Retinol Levels

Five-milliliter blood samples were collected by brachial venipuncture into amber tubes with gel, centrifuged, and stored in dry ice at a temperature of −20 °C for transportation to IMIP’s translational research laboratory. Serum retinol measurement (a dependent variable) was analyzed using high-performance liquid chromatography (HPLC), as recommended by the WHO [9,19], according to the technique established by Furr et al. [20]. The analysis was performed using a Dionex UltiMate 3000 HPLC system (Sunnyvale, CA, USA) with an Acclaim C18 column (5 µm × 4.6 mm × 250 mm) coupled with a C18 Nucleosil guard column, and an injection volume of 20 µL. Detection was performed using a UV detector (Dionex UltiMate 3000 Variable Wavelength Detector) at 325 nm. Retinyl acetate obtained from Sigma was used as a standard, peak retention was 3.6 min, and the mobile phase flow was 1.5 mL/min. The cut-off points used for the classification of retinol levels were: deficient: <0.35 μmo/L; low: ≥0.35 and <0.70 μmol/L; acceptable: ≥0.70 and <1.05 μmol/L; and adequate ≥1.05 μmol/L [14]. VAD was defined as retinol levels <0.70 μmol/L, as recommended by the WHO [9,19]; therefore, the cumulative prevalence of vitamin A levels classified as deficient or low (serum retinol levels <0.70 μmol/L) was adopted as the endpoint for the univariate and multivariate analyses.

### 2.4. Biological, Socioeconomic Data, Obstetric History, and Others

The independent variables (biological and socioeconomic factors, data on obstetric history and prenatal care, and the anthropometric classification of nutritional status) were obtained using a pretested, structured form administered by trained and supervised interviewers. These data included the pregnant woman’s age, per capita monthly income, schooling (evaluated as the number of years of school attendance), number of children ≤5 years of age, number of pregnancies, gestational age in weeks, number of prenatal consultations, whether prenatal care began in the first trimester of pregnancy, use of vitamin supplementation, presence of infection, anemia, and anthropometric indexes classified according to weight and height.

Per capita monthly income was calculated in the number of minimum salaries. Between 2011 and 2012, the value of the minimum salary in Brazilian reais was R$622, which was equivalent to US$342.69 according to the exchange rate at the time (1 US dollar = 1.815 Brazilian reais) [21].

Gestational age was evaluated from ultrasound scans performed in the first trimester of pregnancy. If no scan was available, the date of the woman’s last menstrual period was used. Gestational age was classified at the time of blood sampling as: first trimester (1–13 weeks of pregnancy), second trimester (14–27 weeks of pregnancy), or third trimester (≥28 weeks of pregnancy) [22].

Weight was measured using Plenna^®^ digital scales accurate to 100 grams. Height was measured using a stadiometer (Alturaexata) with graduations in centimeters and millimeters, and approximated readings were obtained for whole or fractional numbers (0 or 0.5 cm). The pregnant women were measured and weighed twice, in their bare feet and with nothing in their hands or pockets. Nutritional status was classified as: underweight, adequate weight, or overweight/obese according to their body mass index adjusted for gestational age [23], in accordance with the recommendations of the Brazilian Ministry of Health [24]. 

Anemia in pregnancy was defined as hemoglobin levels <11 g/L [25], with hemoglobin levels measured using an automated ABX Pentra DF120 analyzer (Horiba, Kyoto, Japan).

The presence of subclinical infection was defined as C-reactive protein (CRP) levels ≥5 mg/L [11], measured by immunoturbidimetry using a clinical chemistry autoanalyzer (Architect c8000, Abbott, Chicago, IL, USA).

### 2.5. Statistical Analysis

The statistical analysis was conducted using the Stata software program, version 12.1. The data on the prevalence of deficient/low and acceptable serum retinol and CRP levels are presented as relative frequencies with their respective 95% confidence intervals (95% CI). The chi-square test was used to assess the association between the classification of serum retinol and CRP levels, with *p*-values <0.05 being considered statistically significant. To calculate the prevalence of VAD and its association with the selected variables, simple and adjusted Poisson regression models were used, and crude and adjusted prevalence ratios were calculated, together with their respective 95% CI. Variables found to be statistically associated with VAD in the univariate model at *p*-values <0.20 were included in the multivariate analysis, with those with *p*-values <0.05 were included in the final model. The statistical significance of each variable was evaluated using the Wald test. Adjusted linear regression models were also constructed to evaluate the correlation between vitamin A, CRP, and gestational age, as well as the correlation between hemoglobin, vitamin A, and gestational age.

### 2.6. Ethical Aspects

This study was conducted in accordance with the Declaration of Helsinki. The internal review board of the Instituto de Medicina Integral Prof. Fernando Figueira approved the protocol of the original study, entitled “The nutritional status of pregnant women: methodological and epidemiological aspects and implications in prenatal care”, under reference number 2471-11. The study was registered under the Certificate of Presentation for Ethical Assessment (CAAE) (reference number 13448413.6.0000.5201) on 20 July 2011. All the women gave their informed consent for inclusion before they participated in the study.

## 3. Results

In this sample of 676 pregnant women, the cumulative prevalence of deficient or low vitamin A levels (<0.70 μmol/L) was 6.2% (95% CI: 4.5–8.3), with the great majority of the women (72.9%; 95% CI: 69.4–76.2) having acceptable levels of serum retinol (≥1.05 μmol/L). Mean serum retinol level was 1.37 μmol/L (SD 0.50). Table 1 shows the minimum and maximum values, as well as the first and third quartiles and the median.

Of the total number of 676 pregnant women, C-reactive protein levels were available for 647. Subclinical infection (CRP ≥5 mg/L) was found to be present in 52.1% of the women (95% CI: 48.0–56.0). As shown in Table 2, there was no evidence of any association between the classification of serum retinol levels and CRP dichotomized into <5 mg/L and ≥5 mg/L (*p* = 0.095).

Table 3 shows VAD as a function of the different characteristics analyzed. The factors found to be significantly associated with VAD in the univariate analysis were: having <12 years of schooling (*p* = 0.041), being in the third trimester of pregnancy (*p* = 0.004), and being anemic (*p* = 0.017). Following adjustment of the model in the multivariate analysis, a prevalence ratio (PR) of 2.85 (95% CI: 1.52–5.36) was found for VAD in the cases in which current gestational age was in the third trimester compared with those in the second trimester (*p* = 0.004). For cases with hemoglobin levels <11 g/dL, the PR was 2.04 (95% CI: 1.15–3.64) for VAD in relation to the group with hemoglobin levels ≥11 g/dL. For the women with fewer than 12 years of schooling, the PR was 1.77 (95% CI: 1.0–3.12) compared to those with ≥12 years of schooling.

Following linear regression analysis, a statistically significant negative correlation was found between vitamin A and gestational age: unadjusted coefficient = −0.013 (95% CI: −0.018–−0.008; *p* < 0.001); adjusted coefficient = −0.013 (95% CI: −0.018–−0.008; *p* < 0.001). On the other hand, no correlation was found between vitamin A and CRP: unadjusted coefficient = 0.0008 (95% CI: −0.005–0.007; *p* = 0.786); adjusted coefficient = −0.001 (95% CI: −0.007–0.004; *p* = 0.646). Regarding hemoglobin, there was no statistically significant correlation either with gestational age or with vitamin A.

## 4. Discussion

The prevalence of VAD of 6.2% in the pregnant women evaluated here represents, according to the WHO criteria, a mild public health problem in this population group receiving prenatal care at a Brazilian regional reference center. This result is in some ways surprising, bearing in mind previous studies conducted with non-pregnant women of reproductive age in Brazil as a whole and in the northeastern region of the country in particular [26,27,28,29]. The difference in the prevalence of VAD between pregnant women, postpartum women, and women of reproductive age is probably attributable to the difference in the cut-off points used: 1.05 μmol/L in previous studies compared to 0.70 μmol/L in the present study. The WHO criteria were used in this study to define VAD as serum retinol levels <0.70 μmol/L, with the degree of public health problem being considered severe when VAD is found in ≥20% of pregnant women, moderate when found in 10–19%, and mild when found in 2–9% [9,14]. Therefore, it would be interesting to apply these criteria in other surveys conducted to evaluate VAD in other disadvantaged areas of the country.

This study is the first to be conducted in Brazil and indeed the first in almost all of Latin America to evaluate vitamin A status in a large group of pregnant women using the most recommended methodology for surveys. The findings reported here are similar to those found in China, where a frequency of VAD of 5.2% was found in a group of 1209 pregnant women evaluated in 150 urban or rural areas between 2013 and 2014 [30]. Compared to the present findings, other studies conducted in developing countries using the cut-off point of <0.70 μmol/L reported much higher prevalence rates of VAD in pregnant women: 24.6% in 3270 pregnant women in Iran [31], 20% in a sample of 80 women in Egypt [32], 18.5% in 200 women in Bangladesh [33], 15.8% in 101 women in Nigeria [34], 13.8% in 738 women in Guinea-Bissau [35], and 10.6% in 160 pregnant adolescents in Venezuela [18]. However, only the sample sizes used in the studies conducted in China, Iran, and Guinea-Bissau were large enough to yield robust data.

In addition to evaluating restricted samples, other studies conducted in Brazil also reported other findings, creating other indicators, i.e. compound and heterogenous markers that could have increased the prevalence of the problem, although these increases were actually the result of distortion effects or biases [16,17,36,37].

Bearing in mind the similarity in the results of the nationwide biochemical survey on the prevalence of VAD conducted in China [30] and the results of the present study, it is important to mention the improvement in the various indicators of life conditions in the population. In fact, Brazil has had one of the fastest processes of nutrition transition in the world, leading the Food and Agriculture Organization (FAO) of the United Nations to remove the country from the World Hunger Map. In addition, protein-energy malnutrition is below endemic levels, with overweight and obesity having become a new epidemic; infant and preschool child mortality rates have decreased; the education level of mothers has increased; and the coverage of basic healthcare actions is practically universal. Although this new scenario has been exhaustively described in the literature [38], unfortunately there are no consistent studies documenting the problem of VAD, particularly in relation to pregnant women. Theoretically, however, it is generally agreed that the situation may have improved. 

Schooling, which has been identified as an important differentiating factor in the results of other surveys on nutrition [30,39,40], was also found to be significantly associated with VAD in the univariate analysis in the present study. Consequently, the prevalence of VAD in the group of women with more than 12 years of schooling was lower. Conversely, in other studies such as that conducted to evaluate VAD in pregnant adolescents in Piauí [37] and another carried out to assess mothers in the state of Pernambuco [41], no statistically significant association was found between VAD and schooling. A fact that needs to be taken into account refers to the rapid nutritional transition occurring in Brazil that differentiates this country from others. Consequently, variables such as family income, education, living conditions, and employment status, previously considered very important, no longer play the same differentiating roles that they did in previous generations.

In relation to the studies conducted in Brazil to evaluate vitamin A status in pregnancy, the majority also included postpartum women [26,27,28,29,42,43,44,45], which may represent a conceptual error in terms of sampling. Issues regarding sample selection and size may also apply to the study conducted with 89 pregnant adolescents conducted in the Brazilian state of Piauí, where VAD was found in 34.8% of the women, consequently constituting a severe public health problem [37]. This very high frequency of VAD compared to the findings of the present study should be examined in the light of the large differences in sample size and the fact that the aforementioned study included only adolescents.

In another two surveys conducted in Brazil, serum retinol levels were not evaluated, with the occurrence of night blindness in pregnant women being the factor taken into account. According to the International Vitamin A Consultative Group (IVACG) [46], night blindness represents a clinical indicator of epidemiological importance. Therefore, in population-based studies, its occurrence in ≥5% of the population would classify VAD as a severe public health problem [46]. Using this criterion, VAD was considered severe in Vale do Jetiquinhonha in the southeastern Brazilian state of Minas Gerais, where night blindness was found in 8.7% of the 92 pregnant women interviewed [16], and in Rio de Janeiro where the prevalence of night blindness was 9.9% in 606 pregnant or postpartum adult women [47].

Serum retinol and its conventional cut-off point for a diagnosis of VAD could be responsible for overestimating the prevalence of vitamin A deficiency in pregnancy, particularly towards the end of pregnancy and in populations with a high prevalence of infection [35]. Thurnham et al. concluded in a meta-analysis that an increase in CRP is associated with a decrease of 25% in serum retinol levels [11]. In the present study, even using CRP values to control for the possible effect of an infectious process, the results were close, with VAD being diagnosed in 4.5% of women with CRP levels <5 mg/L and in 7.4% of those with CRP levels ≥5 mg/L. CRP is an acute phase protein in the inflammatory process, participating predominantly in innate immune response [35,48]. Nonetheless, an increase in CRP levels may occur in many other situations as well as in the presence of infection, including during a normal or physiological pregnancy when levels reflect the adaptations needed to support the fetus. These adaptations occur during the process of maternal immune tolerance, particularly as gestational age increases [49,50].

Therefore, since the infectious process is associated with a transitory reduction in vitamin A biomarkers, the Biomarkers Reflecting Inflammation and Nutritional Determinants of Anemia (BRINDA) project has suggested approaches for correcting for inflammation when estimating VAD in children and women, using both retinol and retinol-binding protein (RBP) [10]. In the present study, adjustment for inflammation, as suggested in the BRINDA project, proved impossible, since the association between serum retinol and CRP was not statistically significant. Likewise, a recent study showed that in non-pregnant women of reproductive age, adjustments for inflammation were precluded due to the inconsistency found in the association between vitamin A biomarkers and inflammation [51]. 

The present results show a greater prevalence of VAD (11.9%) in the third trimester compared to the first and second trimesters of pregnancy, with a negative linear correlation being found between gestational age and serum retinol levels. Other studies have shown similar results, reporting vitamin A deficiency to be more common at the end of pregnancy than at the beginning [18,30,31,35,37,52]. These findings could be explained by the increase in the maternal-fetal transfer of vitamin A due to faster fetal growth towards the end of pregnancy [53]. This may reflect an actual reduction in vitamin A levels [54] and/or a physiological increase in maternal blood volume, which is more pronounced in the final trimester of pregnancy [55], in addition to the immune-mediated inflammatory issue described previously. This suggests a need for prospective cohort studies aimed at identifying specific cut-off points for each trimester of pregnancy in order to establish an accurate prenatal diagnosis of VAD [35].

In relation to reproductive history, no association was found between the number of pregnancies and VAD (PR = 1.64 for the group of women who had had 2–3 pregnancies compared to 1.92 for the group with ≥4 pregnancies). The lack of any statistically significant association was probably due to the small number of women in each of the strata compared; therefore, these results need to be taken merely as an indication. However, likewise, other studies have reported lower serum retinol levels with increased parity [30,34,51,56]. This could be explained by the fact that repeat pregnancies reduce or deplete maternal stocks of nutrients [56]. Conversely, other studies conducted in developing countries have found no correlation between parity and serum retinol levels [31,33,57].

A positive association was found in the present study between the occurrence of anemia and VAD, a finding that is in line with the results of a previous study conducted in China [30]. Vitamin A deficiency is considered one of the causes of anemia, although the pathogenesis of this association remains to be fully clarified. Vitamin A is known to exert an effect on hematopoiesis, increasing immunity to diseases, and thus preventing or reducing anemia resulting from an infection. In addition, it exerts an effect on the modulation of iron metabolism [58]. In a study conducted by Muslimatun et al., vitamin A supplementation during pregnancy was found to increase hemoglobin levels and reduce the occurrence of VAD [59]. A systematic review concluded that antenatal vitamin A supplementation, in addition to iron and folic acid, reduces maternal anemia in populations with a deficiency of vitamin A [60].

In the present study, the reported use of vitamin supplementation had no effect on the results. Nevertheless, no quantitative or qualitative evaluation was made of dietary consumption, which could have had an influence on vitamin A status; therefore, this has to be considered a limitation of the study.

In view of the present findings and the paucity of data on vitamin A nutritional status in pregnant women in Brazil and even in the general population worldwide [61], the need for vitamin A supplementation remains unclear. In fact, supplementation is being introduced in various countries despite the lack of scientific evidence regarding its need and with no knowledge on the prevalence of vitamin A deficiency in that specific region or population group [61]. Therefore, consideration should be given to establishing new guidelines and forms of management for vitamin A supplementation. These should encompass not only pregnant women, the group evaluated in the present study, but also the general population or the most vulnerable biological and social groups such as children [61]. However, there appears to be a consensus on the need for further investigation [61] in special population-based groups in Brazil and in other countries around the world.

## 5. Conclusions

The prevalence of VAD was 6.2% in the pregnant women evaluated here, constituting a mild public health issue and meeting expectations of rates close to those of deficiency control. In the present study, vitamin A deficiency was associated with the third trimester of pregnancy and with maternal anemia. There was no statistically significant association between serum retinol levels and the infectious process evaluated according to levels of CRP; therefore, adjustment for inflammation proved impossible. Consequently, our recommendation is that other similar studies, including an appropriate evaluation of inflammatory markers in accordance with the WHO and BRINDA recommendations, should be conducted in the country based on the hypothesis that the magnitude of the problem has diminished in pregnant women over recent years.

## Figures and Tables

**Table 1:** Distribution of serum retinol levels (μmol/L) in the studied pregnant women. Instituto de Medicina Integral Prof. Fernando Figueira (IMIP), Recife, Pernambuco, Brazil, 2012.

Minimum	Q1	Median	Q3	Maximum
0.26	1.02	1.33	1.67	3.67

Q1 = first quartile; Q3 = third quartile.

**Table 2:** Classification of serum retinol in the studied pregnant women according to C-reactive protein levels. Instituto de Medicina Integral Prof. Fernando Figueira (IMIP), Recife, Pernambuco, Brazil, 2012.

Classification of Serum Retinol Levels (µmol/L)	C-reactive Protein		Total	*p*-Value *
	<5 mg/L	≥5 mg/L		
	*n* (%)	*n* (%)	*n* (%)	
<0.35 (deficient)	0 (0.00)	3 (0.89)	3 (0.46)	0.095 *
≥0.35 and <0.70 (low)	14 (4.52)	22 (6.53)	36 (5.56)	
≥0.70 and <1.05 (acceptable)	75 (24.19)	63 (18.69)	138 (21.33)	
≥1.05 (adequate)	221 (71.29)	249 (73.89)	470 (72.64)	
Total	310 (100.00)	337 (100.00)	647 (100.00)	

* Chi-square test.

**Table 3:** Prevalence of vitamin A deficiency according to biological characteristics, obstetric history, prenatal care, and other factors in a group of pregnant women receiving care at the Instituto de Medicina Integral Prof. Fernando Figueira (IMIP), Recife, Pernambuco, Brazil, 2012.

Characteristics	Prevalence of Vitamin A Deficiency					
	Sample					
			Crude PR		Adjusted PR	
	*n* = 676 *	*n* (%)		*p*-Value		*p*-Value
	*n* (%)		(95% CI)		(95% CI)	
Per capita Income in Minimum Salaries **				0.306		-
<½ minimum salary	123 (18.2)	11 (8.9)	1.85 (0.84–4.09)		-	
½ to 1 minimum salary	303 (44.9)	19 (6.3)	1.30 (0.64–2.63)		-	
>1 minimum salary	249 (36.9)	12 (4.8)	1		-	
**Years of Schooling**				0.041		0.051
≥12 years	438 (64.8)	21 (4.8)	1		1	
<12 years	238 (35.2)	21 (8.8)	1.84 (1.02–3.30)		1.77 (1.0–3.12)	
**Woman’s Age**				0.704		-
≤19 years	54 (8.0)	4 (7.4)	1.21 (0.45–3.27)		-	
≥20 years	622 (92.0)	38 (6.1)	1		-	
**Number of Children ≤5 Years of Age**				0.594		-
None	498 (73.7)	33 (6.6)	1.46 (0.66–3.23)		-	
1	154 (22.8)	7 (4.5)	1		-	
≥2	24 (3.6)	2 (8.3)	1.83 (0.40–8.32)		-	
**Number of Pregnancies Including Current Pregnancy**				0.247		-
1	292 (43.2)	13 (4.4)	1		-	
2 or 3	314 (46.4)	23 (7.3)	1.64 (0.85–3.19)		-	
≥4	70 (10.4)	6 (8.6)	1.92 (0.76–4.89)		-	
**Current Trimester of Pregnancy**				0.004		0.004
First	116 (17.5)	7 (6.0)	1.45 (0.61–3.45)		1.48 (0.62–3.52)	
Second	386 (58.4)	16 (4.1)	1		1	
Third	159 (24.1)	19 (11.9)	2.88 (1.52–5.46)		2.85 (1.52–5.36)	
**Number of Prenatal Consultations**				0.477		-
1–5	653 (97.0)	40 (6.1)	1		-	
≥6	20 (3.0)	2 (10.0)	1.63 (0.42–6.29)		-	
**Prenatal Care Initiated in the First Trimester**				0.077		0.406
Yes	465 (69.4)	24 (5.2)	1		1	
No	205 (30.6)	18 (8.8)	1.70 (0.94–3.07)		1.29 (0.71–2.33)	
**Use of Vitamin Supplementation**				0.831		-
Yes	337 (50.4)	19 (5.6)	1		-	
No	332 (49.6)	20 (6.0)	1.07 (0.58–1.97)		-	
**C-Reactive Protein**				0.126		0.093
≥5 mg/L	337 (52.1)	25 (7.4)	1.64 (0.87–3.10)		1.72 (0.91–3.22)	
<5 mg/L	310 (47.9)	14 (4.5)	1		1	
**Hemoglobin**				0.017		0.015
<11 g/dL	180 (26.8)	18 (10.0)	2.04 (1.14–3.68)		2.04 (1.15–3.64)	
≥11 g/dL	491 (73.2)	24 (4.9)	1		1	
**Nutritional Status**				0.359		-
Underweight	81 (13.2)	3 (3.7)	1		-	
Adequate weight	240 (39.2)	18 (7.5)	2.02 (0.61–6.70)		-	
Overweight/obese	291 (47.6)	15 (5.2)	1.39 (0.41–4.69)		-	

* Sample size varies due to missing data in some cases; ** Minimum salary was US$ 342.69 at the time of admission to the study. CI: confidence interval.
